# Medical errors; causes, consequences, emotional response and resulting behavioral change

**DOI:** 10.12669/pjms.323.9701

**Published:** 2016

**Authors:** Attia Bari, Rehan Ahmed Khan, Ahsan Waheed Rathore

**Affiliations:** 1 Dr. Attia Bari, MBBS, DCH, MCPS, FCPS. Associate Professor of Pediatric Medicine, Department of Pediatric Medicine, The Children’s Hospital and The Institute of Child Health, Lahore, Pakistan; 2 Rehan Ahmed Khan, MBBS, FCPS, FRCS, JM-HPE, MSc HPE. Associate Professor of Surgery, Islamic International Medical College, Riphah University, Pakistan; 3 Ahsan Waheed Rathore, MBBS, DCH, MRCP, MRCPCH, FRCP. Professor of Pediatric Medicine, Department of Pediatric Medicine, The Children’s Hospital and The Institute of Child Health, Lahore, Pakistan

**Keywords:** Medical errors, Emotional response, Error disclosure

## Abstract

**Objective:**

To determine the causes of medical errors, the emotional and behavioral response of pediatric medicine residents to their medical errors and to determine their behavior change affecting their future training.

**Methods:**

One hundred thirty postgraduate residents were included in the study. Residents were asked to complete questionnaire about their errors and responses to their errors in three domains: emotional response, learning behavior and disclosure of the error. The names of the participants were kept confidential. Data was analyzed using SPSS version 20.

**Results:**

A total of 130 residents were included. Majority 128(98.5%) of these described some form of error. Serious errors that occurred were 24(19%), 63(48%) minor, 24(19%) near misses,2(2%) never encountered an error and 17(12%) did not mention type of error but mentioned causes and consequences. Only 73(57%) residents disclosed medical errors to their senior physician but disclosure to patient’s family was negligible 15(11%). Fatigue due to long duty hours 85(65%), inadequate experience 66(52%), inadequate supervision 58(48%) and complex case 58(45%) were common causes of medical errors. Negative emotions were common and were significantly associated with lack of knowledge (p=0.001), missing warning signs (p=<0.001), not seeking advice (p=0.003) and procedural complications (p=0.001). Medical errors had significant impact on resident’s behavior; 119(93%) residents became more careful, increased advice seeking from seniors 109(86%) and 109(86%) started paying more attention to details. Intrinsic causes of errors were significantly associated with increased information seeking behavior and vigilance (p=0.003) and (p=0.01) respectively.

**Conclusion:**

Medical errors committed by residents have inadequate disclosure to senior physicians and result in negative emotions but there was positive change in their behavior, which resulted in improvement in their future training and patient care.

## INTRODUCTION

“To err is human”.^1^ Medical errors are inevitable and can have a disastrous effect on patient, treating doctor, nurses and the institution as well.^2^,^3^ Building a safe health care system means designing processes of care to ensure that patient are safe from accidental injury. A report on safety in health care by Institute of Medicine publication, To Err is human, focused attention on this problem, particularly its conclusion that every year more Americans die as a result of medical errors than deaths from automobile accidents and indicated that there were up to 98,000 deaths per year because of medical errors.^4^

Virtually all doctors have made mistakes but they often don’t tell patients or families about them. In clinical practice human errors are commonbut they are generally underreported.^5^ As a result of this underreporting very little is known about the causes and consequences of medical errors. Moreover facing to a medical error is never easy and hence it is not disclosed.^6^ Often it is difficult to recognize one’s mistake, but it is necessary to face the situation and try to learn from it so that future errors can be prevented. Identifying the risk factors for medical errors is crucial first step towards its prevention and is important goal of quality care assurance.^7^

Self-perceived medical errors are common among doctors and are associated with subsequent personal distress. As a consequence of medical error health care providers at all training levels experience feelings of guilt, disappointment, fear and sense of inadequacy of varying degree.^3^,^8^ Impact of medical error on health care provider is a vital area deserving attention. Residents are vulnerable population whose early experience shapes their future behavior. Residency period plays a critical role in defining physicians’ future practice and responses to medical error.^9^,^10^ Post-graduate residents and house officers often choose not to disclose their mistakes to the attending physician. Trainees who have accepted responsibility for the mistake and have discussed it were more likely to report constructive changes in practice. Residents were less likely to make constructive changes if they attributed the mistake to job overload.^6^

Residents need special attention because behaviors learnt early in practice are more likely to persist in their later professional carrier.^9^ We planned this study to learn how medical errors relate to subsequent changes in practice.

## METHODS

This was a prospective hospital based cross sectional study, conducted at Children’s Hospital and Institute of Child Health Lahore which is a tertiary care hospital with 650 beds and around 250 postgraduate residents. The study population included pediatric medicine residents. The study proposal received approval from hospital ethical committee. After taking permission from the author Hobgood,^8^ her Questionnaire was adopted. We pilot tested the questionnaire on a sample of 25 for reliability that was 0.852 Cronbach’s Alpha. Questionnaire was distributed to 150 pediatric medicine postgraduate residents and 130 residents returned the questionnaire proforma with a response rate of 87%. The survey was anonymous as residents were asked to complete the questionnaire without indicating their names. We asked the residents to answer the questionnaire by recalling the most significant error encountered during their residency period using the definitions for key terms. These were medical error, serious error, minor error and near misses. These were defined as follows: **Medical error**:The failure of a planned action to be completed as intended or the use of a wrong plan to achieve an aim. **Serious error**: An error that causes permanent injury or transient but life threatening harm.**Minor error**: An error that causes harm that is neither permanent nor potentially life threatening. **Near misses**: An error that could have caused harm but did not either by chance or timely intervention,^11^ provided at the beginning of the questionnaire. Our study explored the residents’ perception of cause of medical error, their responses to these errors, its disclosure and the effect of that error (constructive or defensive) on their behavior. Residents were asked to complete questionnaire about their errors and responses to their errors in three domains: emotional response, learning behavior and disclosure of the error. The three distinctive behavioral changes were information seeking, vigilance and defensive practice. We used 5 point Likert scales (1= Strongly agree, 2= Agree, 3= Neutral, 4= Disagree, 5= Strongly disagree) to assess residents response to errors. Demographic questions included participant’s age, sex and year of residency. The names of the participants were kept confidential.

Answers from the questionnaire were entered into Statistical Package for Social Sciences (SPSS) version 20 software for analysis. Descriptive statistics included mean and standard deviations for continuous variables and frequencies and percentages for categorical variables. The chi-square test was used for statistical analysis. A p value of <0.05 was considered statistically significant.

## RESULTS

Postgraduate residents reported that errors occur frequently among admitted patients and had both intrinsic and extrinsic attribution of errors. Of the 130 participants providing error data most participants 128(98.5%) had encountered a medical error, only 2(1.5%) reported no error involvement and 17(13%) mentioned cause and effect of error but did not specify the type of error.Table-I. The age distribution for the entire participants was 28±1.9 years. Demographic characteristics of the participants are shown in Table-II.

**Table-I:** Types of medical errors.

*Medical Errors*	*Percentage*
Serious medical error	18%
Minor medical error	48%
Near misses	19%
Never encountered medical error	2%
Not mentioned type of error but mentioned cause and effect	13%

**Table-II:** Characteristics of residents who participated in the study.

*Category*	*Total*
	n =130 (100%)
*Age* Mean 25-30 years 31-35 years > 35 years	28±1.98 Years 118 (90%) 11 (9.2%) 01 (0.8%)
*Sex (M:F)* Male Female	59 (45%) 71 (55%)
*Year of Post Graduate Training* 1st Year 2nd Year 3rd Year 4th Year	24 (19%) 26 (20%) 46 (35%) 34 (26%)

The intrinsic and extrinsic attributions of medical errors are shown in Table-III. The common intrinsic attribution that residents narrated was fatigue due to long duty hours 85(65%), inadequate experience 66(52%), followed by missing of warning signs 51(40%). With respect to extrinsic attribution, 81(63%) reported having other things to take care of, 61(48%) identified that case was complex and 58(45%) narrated inadequate supervision by the senior was the factor. Residents who attribute their error due to fatigue or job overload did not show any constructive change in their behavior. (Table-IV).

**Table-III:** Causes of medical errors and error disclosure.

*Cause of the medical error*	*(Who agreed/strongly agreed) N (%)*
*Intrinsic* I did not have enough experience I did not possess enough knowledge I missed the warning signs There was faulty communication I was tired/ fatigued due to long duty hours I did not ask for advice from senior I hesitated too long	66 (52%) 51 (40%) 51 (40%) 46 (36%) 85 (66%) 27 (21%) 13 (10%)
*Extrinsic* I had many other things to take care of The case was very complex It was an atypical presentation There was inadequate supervision There was a procedural complication Lab report was wrong so resulted in misjudgment	81 (63%) 61 (48%) 57 (45%) 58 (45%) 37 (29%) 24 (19%)
*Disclosure* To none due to fear / guilt/ embarrassment To my colleague present with me on duty To my close friend/ spouse To my senior / physician involved in the case Discussed with some other senior who is not involved in that case To patient family or patient	27 (21%) 90 (70%) 74 (58%) 73 (57%) 44 (34%) 15 (11%)

**Table-IV:** Association of causes of medical errors with behavioral response.

*Behavioral Response*	*Intrinsic Causes of Errors p value*			*Extrinsic causes of Errors p value*		
	*Did not possess enough Knowledge*	*Missed Warning Signs*	*Fatigued due to long duty*	*Many other things to take care of*	*Atypical Presentation*	*Procedural Complication*
*Information Seeking*					
Seek more advice from seniors	0.003	0.028	0.141	0.691	0.142	0.017
Ask supervision more often	0.014	0.164	0.807	0.865	0.142	0.103
Read more about cases	0.042	0.143	0.375	0.854	0.245	0.266
*Vigilance*					
Pay more attention to details	0.016	0.004	0.648	0.966	0.007	<0.001
Use evidence based medicine	0.003	0.753	0.637	0.737	0.395	0.031
*Defensive*					
Order more test	0.076	0.517	0.963	0.310	0.015	0.341
Keep errors to myself	0.024	0.217	0.507	0.397	0.763	0.041
Avoid similar patients	0.012	0.171	0.182	0.172	<0.001	<0.001
See fewer patients	0.026	0.164	0.213	0.640	0.131	0.010

All 128(100%) residents who encountered an error reported experiencing some negative emotions as a result of their error. Most 89(70%) experienced sorrows, 88(69%) guilt, 85(66%) emotional distress and 51(40%) inadequacy. (Table-V) Negative emotions were significantly associated with intrinsic causes like lack of knowledge (p=0.001), missing warning signs (p=<0.001) and not seeking advice (p=0.003). Residents who mentioned extrinsic attribution to the error also reported to have negative emotions that were significantly associated with procedural complication (p=0.001) and atypical presentation (p= 0.018).

**Table-V:** Emotional and behavioral responses to medical errors.

*Responses*	*(Who agreed/strongly agreed) n (%)*
*Emotional Response*	
*Negative Emotions*	
In reaction to error I felt a lot of:
Emotional distress	85 (66%)
Sorrow	89 (70%)
Guilt	88 (69%)
Inadequacy	51 (40%)
Frustration	49 (38%)
Fear	38 (30%)
OR	
It was not my fault	20 (16%)
*Behavioral Response*
*1. Increased Information Seeking*
• Always seek more advice from senior staff	109 (85%)
• Seek more advice from peers	87 (68%)
• Ask supervision more often	96 (76%)
• Ask for more literature reference	86 (67%)
• I read more from the book about the cases	64 (50%)
*2. Increased vigilance*
• Pay more attention to details of patient	109 (85%)
• Use more evidence based medicine	99 (77%)
• Personally confirm data	88 (69%)
• Trust others’ judgment less	69 (53%)
• Became more careful	119 (93%)
• Always recheck the lab report when in doubt	95 (74%)
*3. Increased defensive attitude*
• Order more tests	40 (31%)
• Keep errors to myself more often	29 (23%)
• Avoid similar patients	14 (11%)
• See fewer patients	20 (16%)

Medical errors resulted in significant change in resident’s learning behaviors. Eighty five percent sought more advice from seniors, 96(76%) started asking for supervision more often. Increased vigilance was a significant behavior change as 119(93%) became more careful, 109(85%) reported paying more attentions to the details of the case, and 99(77%) using evidence based medicine. Intrinsic causes of errors like lack of knowledge and missing warning signs were significantly associated with increased information seeking behavior and vigilance (p=0.003) and (p=0.01) respectively.(Table-IV) Only few residents reported increased defensive attitude: 14(11%) reported avoiding similar patients, 40(31%) ordering more test and only 20(16%) reported seeing fewer patients. (Table-V)

As far disclosure was concerned most respondents’ 103(80%) disclosed the medical error to someone. (Table-III)Those who discussed their error with the senior physician involved in the case were only 73(57%), disclosure to none was 27(21%) and least number of residents 15(11%) disclosed the error to the patient’s family. Not disclosing the error to anyone was significantly associated with intrinsic causes like not possessing enough knowledge (p=0.001), not having enough experience (p=0.001), missing warning signs (p=0.01) and extrinsic cause of procedural complication (p= 0.018). Error disclosure to senior was significantly associated with atypical presentation (p=0.037), complex case (p=0.015), not possessing enough knowledge (p=0.024). Those who did not disclose their errors showed more defensive attitude with seeing fewer patient and avoiding similar patients (p=<0.001), ordering more tests (p=0.045) and keeping the errors to themselves (p=0.024).

## DISCUSSION

To improve patient safety, it is necessary to know about the causes, frequency and seriousness of medical errors.^12^ Residents make medical errors in every clinical context. Residency is a time of learning and resident learns to acquire increasing responsibilities of clinical decision-making and professional development. Future clinical practice is affected by the behavioral response to their errors. Understanding the effect of medical errors on residents’ behavior is critical and teaching faculty must understand how resident respond to their errors. Residents can be helped by encouraging them to develop positive error management strategies.

In our study 18% residents reported serious errors and 48% minor errors. Similar findings were noted in different studies, which showed major errors resulting in deaths in 31%, 34%and 39% respectively.^6^,^13^,^14^

Among trainees subsequent personal emotional distress is associated with self perceived medical errors.^13^ The results of our study showed that errors that occurred during residency training have substantial negative emotional impact which is consistent with the study done by Hobgood,^8^ West^13^,^14^ and result in learning behavior change. The resulting negative emotions due to error were guilt, emotional distress sorrow and inadequacy. These negative emotions were significantly associated with lack of knowledge, missing warning signs, not seeking advice and procedural complication.

Hospitals’ functioning is round the clock, post-graduate residents work for long hours. They are often sleep deprived and fatigued. Sleepiness and fatigue affect patient’s safety.^14^,^15^ In our study majority 66% of residents reported that fatigue or tiredness due to long duty hours was the cause of their medical error. Lack of experience, inadequate supervision by seniors was also reported by 52% and 45% respectively and are similar to study by Singh and Hobgood.^5^,^8^ All these factors need to be addressed by hospital administration. Poor communication is an important cause of adverse events in health care system, resulting in medical errors that range from delay in treatment to wrong site surgery. In our study 36% of postgraduate trainees reported faulty communication as a cause of error. Routine team checklist briefing has a positive effect on team communication and teamwork.

Unfortunately very few residents have been taught how to disclose the error and majority do not have proper experience of disclosing an error. They generally use the informal way of disclosure such as telling to someone they trust or not fearful of.^16^ Same was reported in our study in which 80% residents informally discussed their errors. Trainees often choose not to disclose their medical errors to their senior physicians or supervisors.^3^,^17^ Our recent data suggest that professional modelling of error acknowledgement and discussion of errors needs more attention as only 57% of residents in our study discussed their errors with their senior or supervisors, comparable with the study published in JAMA in which 54% discussed their errors with seniors.^6^

Patients and their families wish to be informed immediately about the medical errors that occur.^18^ However, the disclosure to patient is often limited.^19^,^20^ Our results highlighted that disclosing medical error to family members is a challenging task. Consistent with the finding of Wu et al.^6^ which showed disclosure to patient in 24%, only 11% of our resident disclosed their error to the family which may be due to their concept that patient’s family would not understand or blame them.

Open communication about errors presents huge challenges for residents. Our medical profession should develop disclosure guidelines to help the treating physicians and pediatric residency training should include formal instructions in error disclosure. We don’t have well-established hospital incident report system and medical errors in pediatric patients are significantly underreported. Information in incident reports is not a representation of actual medical errors committed in pediatric hospitals.^21^ Establishing a proper incident reporting system can lead to more error reporting by doctors.

Very little is known about what and how the residents are taught about medical errors.^22^ Although in our health care system there is no proper teaching or lectures about medical errors but mortality and morbidity conference definitely help residents to learn from joint discussions of mistakes.

Senior health care professionals must be supportive and nonjudgmental of their residents’ when medical errors take place. Discussing one’s own error experience can help to reduce the resident’s sense of isolation and guilt.^2^

## CONCLUSION

Residents encounter medical errors at all levels of training. Fatigue due to long duty hours, lack of experience, job over load and inadequate supervision by senior were major causes of these errors. Medical errors committed by residents have inadequate disclosure to senior physicians. Errors resulted in negative emotions but there was a positive change in their behavior, which resulted in improvement in their future training and patient care. There is a need for close monitoring of postgraduate training program, adequate round the clock senior supervision, assessment of their professional competence on regular basis, regularization of duty hours to prevent fatigue and also legal protection for doctors and patients.
